# The Importance of Intrinsically Disordered Segments of Cardiac Troponin in Modulating Function by Phosphorylation and Disease-Causing Mutations

**DOI:** 10.3389/fphys.2016.00508

**Published:** 2016-11-02

**Authors:** Maria Papadaki, Steven B. Marston

**Affiliations:** ^1^Department of Cell and Molecular Physiology, Loyola University of ChicagoMaywood, IL, USA; ^2^Myocardial Function, National Heart and Lung Institute, Imperial College LondonLondon, UK

**Keywords:** cardiac troponin, molecular dynamics, intrinsic disorder, phosphorylation, cardiomyopathy

## Abstract

Troponin plays a central role in regulation of muscle contraction. It is the Ca^2+^ switch of striated muscles including the heart and in the cardiac muscle it is physiologically modulated by PKA-dependent phosphorylation at Ser22 and 23. Many cardiomyopathy-related mutations affect Ca^2+^ regulation and/or disrupt the relationship between Ca^2+^ binding and phosphorylation. Unlike the mechanism of heart activation, the modulation of Ca^2+^-sensitivity by phosphorylation of the cardiac specific N-terminal segment of TnI (1–30) is structurally subtle and has proven hard to investigate. The crystal structure of cardiac troponin describes only the relatively stable core of the molecule and the crucial mobile parts of the molecule are missing including TnI C-terminal region, TnI (1–30), TnI (134–149) (“inhibitory” peptide) and the C-terminal 28 amino acids of TnT that are intrinsically disordered. Recent studies have been performed to answer this matter by building structural models of cardiac troponin in phosphorylated and dephosphorylated states based on peptide NMR studies. Now these have been updated by more recent concepts derived from molecular dynamic simulations treating troponin as a dynamic structure. The emerging model confirms the stable core structure of troponin and the mobile structure of the intrinsically disordered segments. We will discuss how we can describe these segments in terms of dynamic transitions between a small number of states, with the probability distributions being altered by phosphorylation and by HCM or DCM-related mutations that can explain how Ca^2+^-sensitivity is modulated by phosphorylation and the effects of mutations.

## Introduction

Since its discovery by Ebashi and Kodama (1965), troponin has been a molecule that never ceases to be studied, due to its central role in muscle contraction and regulation. In heart muscle, troponin has a dual function, both to switch contraction on and off in response to Ca^2+^ and to modulate Ca^2+^-sensitivity and the rate of relaxation in response to adrenaline, a process that is disrupted in cardiomyopathy (Solaro et al., 2008; Messer and Marston, 2014). To properly understand these functions of troponin and the mechanisms behind them, it is important to establish structure-function relationships. Recently, cardiac troponin has been recognized as a “dynamic” molecule, containing intrinsically disordered regions (Colson et al., 2012; Hwang et al., 2014). We are particularly interested in these regions, their relationship to troponin's function and how they change due to phosphorylation and mutations. Ultimately, this information can be used for the treatment of cardiomyopathy, by designing drugs that will target these dynamic transitions.

## The regulation of contraction by troponin

**Figure 1:**
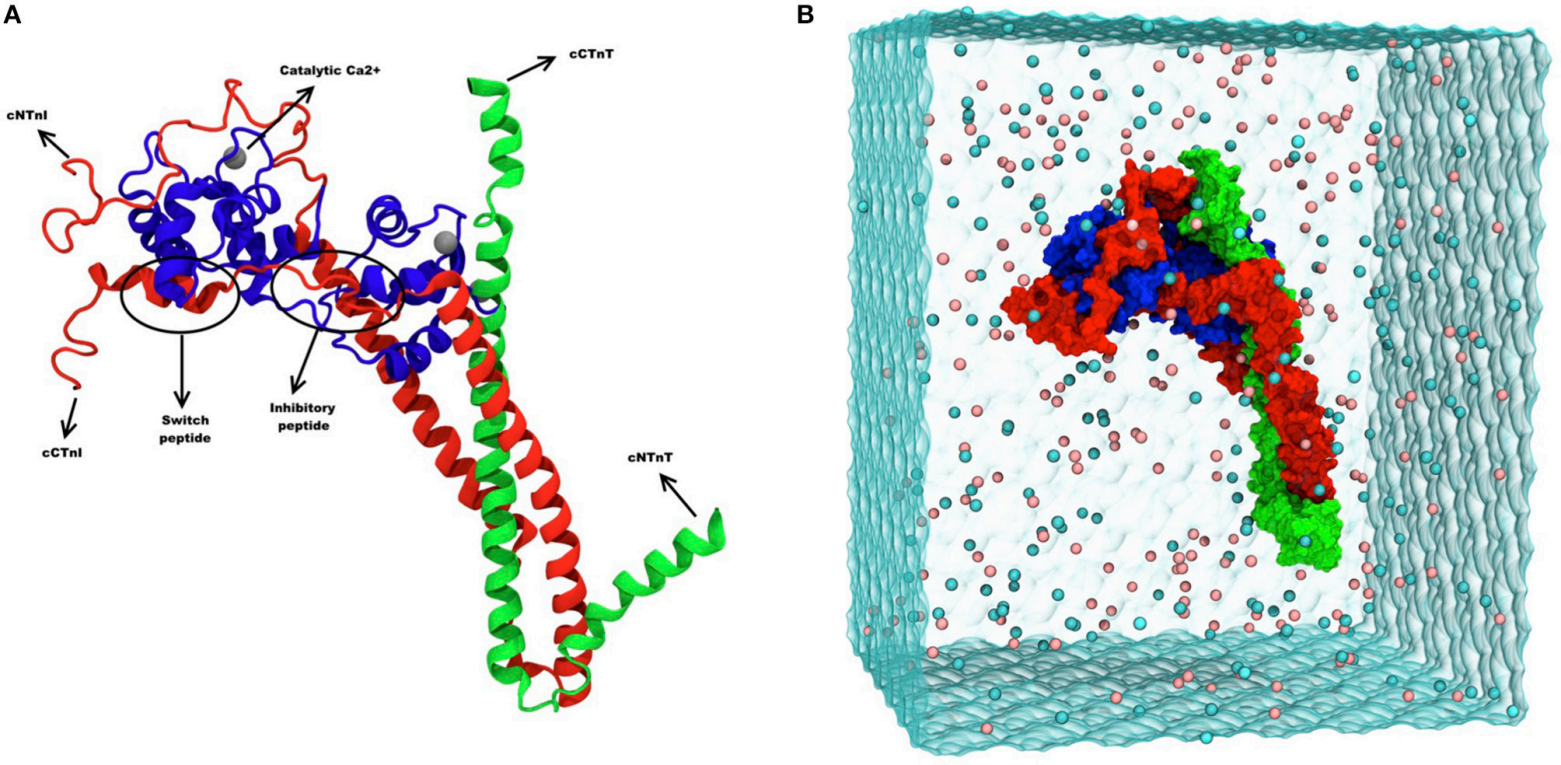
**(A)** Ribbon diagram of troponin and its different subunits. Blue represents TnC (1–161), red represents TnI (1–171) and green represents TnT (212–298). Catalytic and structural Ca^2+^ are shown as gray spheres, and the inhibitory and the switch peptide of TnI are indicated. **(B)** Snapshot of troponin MD simulation, showing troponin surface rendering in a solvent box. Blue represents TnC (1–161), red represents TnI (1–171) and green represents TnT (212–298). The image was adapted from Zamora et al. (2016a) with permission from the PCCP Owner Societies.

The early studies on troponin used biochemical and biophysical methods to measure troponin's activity switching and identify protein-protein interactions involved. It soon became apparent that troponin-regulated thin filaments are a very complex system. The thin filament is a multiprotein complex with a repeating unit comprised of 24 proteins [14 actin, 2 tropomyosin dimers, 2 troponin I (TnI), 2 troponin T (TnT), and 2 troponin C (TnC)]. The troponin molecule complex is shown in Figure 1A. The thin filament can contain up to 30 such units in a system that allows Ca^2+^ binding to TnC to control myosin binding to actin, allowing contraction (Gordon et al., 2000). As expected for a switch, the whole system is dynamic and the properties of the switch are modified by cellular signaling systems, notably phosphorylation of TnI by PKA. Whilst thin filaments can be described in terms of the protein interactions involved (Farah and Reinach, 1995), or the transitions between states (Maytum et al., 1999), a complete structure of the thin filament remains elusive.

The structure of the actin filament has been solved to high resolution (Oda et al., 2009), as well as tropomyosin by X-ray diffraction (Brown et al., 2005) and the complex of tropomyosin on actin has been accurately deduced from electron micrographs (Moore et al., 2016). Troponin was the last component to be solved by crystallography, reflecting both its heterotrimeric nature and its inherent flexibility (Takeda et al., 2003; Vinogradova et al., 2005). The troponin crystal structure is incomplete, however, and does not include some of the most functionally interesting parts of the complex.

These structural studies suffer from a critical defect: they only provide structures that are frozen in a particular state that may or may not correspond to one of the functional states defined by biochemistry, yet troponin is a highly dynamic molecule that easily flips between activity states. Current structures do not resolve several segments of troponin that are potentially involved in regulation including the inhibitory (138–147) and switch (148–154) regions of TnI as well as the C-terminal mobile domain (164–210) and the C-terminal 18 amino acids of TnT. Thus, for understanding troponin function we need to define the structure of the whole molecule and take into account its dynamic nature. Recent concepts describe that order-disorder transitions in the dynamic regions of troponin are important for muscle contraction regulation (Metskas and Rhoades, 2016).

The C-terminal mobile peptide of skeletal TnI was the first one to be identified as a dynamic peptide on troponin and its dynamic structure gives kinetic activity advantage for binding to actin (Blumenschein et al., 2006; Hoffman et al., 2006). The dynamic segments of cardiac troponin were explicitly defined by Tobacman using isotope exchange methods (Kowlessur and Tobacman, 2012), whereas fluorescent anisotropy experiments on cardiac TnI provided experimental evidence on the existence of these dynamic domains (Zhou et al., 2012). The dynamic regions of TnI vary between troponin isoforms, with skeletal troponin having a higher level of disorder. In addition, cardiac TnI contains a disordered region absent in the skeletal isoform, the N-terminal extension containing the phosphorylatable Ser22 and 23 (Hoffman and Sykes, 2008). Phosphorylation at Ser22 and 23 causes a 2-3-fold change in Ca^2+^-sensitivity, which has a major physiological impact on cardiac function.

## Recent studies of cardiac troponin regulation

Baryshnikova demonstrated that phosphorylation of TnI Ser22 and 23 modulates the overall affinity of the N-terminal lobe of TnC to TnI (147–163) rather than the Ca^2+^-affinity (Baryshnikova et al., 2008). A study of the interaction of the native and phosphorylated TnI (1–30) in isolation led to a model for the differential docking of this peptide on TnC in native and phosphorylated states (Howarth et al., 2007), but more recently the question was addressed in a model-free study using whole TnC and TnI (1–73) (Hwang et al., 2014). TnI (1–37) segment, described as intrinsically disordered, interacts electrostatically with the N-terminal lobe of TnC, whilst TnI (41–67) forms a helix that interacts with a hydrophobic patch in the C-terminal TnC lobe, as found in the crystal structure. This study proposed that interactions of TnI (1–30) with the TnC N-terminal lobe stabilized the position of the N-terminal lobe relative to the rest of troponin and that this positioning indirectly affects Ca^2+^ and TnI (148–158) affinity. Phosphorylation disrupts the interactions and the loss of positioning leads to changes in Ca^2+^-sensitivity. Disorder in proteins is currently studied using molecular dynamics (MD) simulations in combination with structural studies, such as NMR.

Molecular dynamics (MD) is a computational technique whereby proteins can be viewed as moving objects rather than stable peptides. Thus, intrinsically disordered peptides can be analyzed in terms of their dynamic, as well as their static properties. The idea behind MD is to calculate the energy of a system as a function of the position of its atoms. MD simulations can help us understand biological functions of proteins, such as conformational changes caused by interactions with other proteins or ligands. Additional properties can be measured, for example flexibility of proteins traditionally measured in nanosecond (ns) timescales, although biological events happen within microseconds (μs).

Recent technical developments have applied this computational method to cardiac troponin. A seminal MD study simulated Ca^2+^ binding to the TnC N-terminal domain over a μs timescale, much longer that commonly used (Lindert et al., 2012). This simulation showed the dynamics of opening and closing of the hydrophobic patch upon Ca^2+^ binding predicted by NMR studies, whilst also emphasizing the need for extended simulations to allow time for all possible conformations to be explored (Lindert et al., 2012). This highlights a central challenge of MD studies: the immense amount of time and computing power needed for an extended simulation must be balanced by the need to obtain results in a meaningful timescale. Moreover the protein investigated must be placed in a virtual solvent box big enough to avoid any contacts with the box sides. The larger the system studied the greater the problems become, however MD simulations of troponin (about 500,000 atoms including solvent) have now been carried out by two groups.

Molecular dynamics (MD) simulations of the cardiac troponin were performed in a study by Cheng et al. (2014). In that study, the troponin complex structure [TnI (1–172), TnC (1–161), TnT (236–285)] was modeled and the phosphomimetic mutations S23D/S24D were introduced to study the effect of phosphorylation and also the effect of disease-related mutations. The length of simulation was limited to 3 × 150 ns (Cheng et al., 2015). The more recent MD simulation study by Zamora et al provides an even more complete model of human cardiac troponin sequence (Figure 1B), including the C-terminal peptide of TnT not present in previous studies. Multiple 750 ns simulations, totaling over 10 μs, were performed and the size of the box simulated has been increased to 25Å to avoid virtual self-association (Zamora et al., 2016a).

## Insights for troponin regulatory mechanism

Recent studies have come to the conclusion that cardiac troponin contains intrinsically disordered regions, and one of them is the TnI (1–30), which contains the phosphorylatable Ser22 and 23. In fact, being disordered is significant for the phosphorylation itself and it has been found that the majority of proteins that get phosphorylated in nature have intrinsically disordered domains close to the phosphorylation site (Iakoucheva et al., 2004). Using MD simulations to study the effects of mutations or phosphorylation is therefore a suitable approach, because the disordered regions are included in the model. MD provides the opportunity to look at the overall stability of the protein by calculating the Root Mean Square Fluctuation (RMSF) and any interaction between the subunits can be measured.

In that respect, it is important to establish which interactions are relevant to troponin's function. Both Zamora's and Cheng's MD studies agree that the order-disorder transition associated with phosphorylation proposed by Hwang et al is unlikely since both states are disordered. Distances between Ca^2+^ and coordinating residues within the Ca^2+^ binding loop are almost always measured, because direct comparisons with biochemical measurements can be made. The MD studies of Cheng and Zamora indicate that measuring the distances between the TnC Ser69 gamma Oxygen (GO) and Ca^2+^ would give information about the Ca^2+^-sensitivity change. Ser69 GO was found in two different conformations: at a high distance and a low distance. Cheng et al observed the low distance conformation only 10% of the time, while Zamora et al found the opposite; the Ser69 GO is located within the coordination sphere (~3Å) most of the time but flips to a longer distance (~6Å) outside the Ca^2+^ coordination sphere occasionally, where it can stay for ~5% of the time. Phosphorylation doubles the time spent at the long distance, which may explain the Ca^2+^-sensitivity change upon phosphorylation. The difference is ascribed by Zamora et al to the shorter simulation times in the Cheng et al study that have only sampled the longer distance state. In addition, it is thought that the small box size used may result in periodic artifacts mostly due to the L shaped configuration of the starting molecule and the hinge motion that elongates the molecule, increasing the probability of self-association. The study of Zamora et al used real phosphorylation in Ser22 and 23 whereas the study of Cheng et al used phosphomimetic mutations S23/S24D, although there are no structural or physiological differences between the effects of phosphorylation and phosphomimetic mutations in the myofilament (Finley et al., 1999; Mamidi et al., 2012; Rao et al., 2014). Using S23/24D allowed Cheng et al to make direct comparisons between MD simulations and physiological data.

The contacts between the intrinsically disordered segments and the troponin core are interesting since they are involved in the mechanism of regulation by troponin (Cheng and Regnier, 2016). In order for the opening of the hydrophobic cleft upon Ca^2+^ binding to occur, TnC must interact with the TnI switch peptide (148–154). The N-terminal TnC-TnI (1–30) interaction is also crucial for the phosphorylation signal to modulate Ca^2+^-sensitivity. Finally, the disordered C-terminus of TnT (212–298), which has not previously been simulated, maybe involved in these interactions.

In their MD simulations, Cheng et al observed that phosphorylation decreased the overall stability of troponin as determined by RMSF plots and changed the interactions between TnC and TnI. More specifically, they found increased intrasubunit interactions between the N-terminal and the inhibitory TnI peptide, which are not present in the unphosphorylated structure. In contradiction to this study, the study by Zamora et al showed that phosphorylation does not affect overall stability of any part of the troponin complex and that phosphorylation changes the interactions between the intrinsically disordered segments and TnC. Moreover, the C-terminus of TnT has proven to make a significant difference to the analysis since it interacts with both TnC N-terminal domain and the TnI N-terminal peptide.

In essence, Zamora's analysis indicates that phosphorylation never induces new interactions between the subunits but results in a number of subtle changes in the dynamics of existing interactions. A recent study supports this finding, as the TnI mutation R145W causing restrictive cardiomyopathy reduces the interaction frequencies between TnC and TnI leading to blunting of the adrenergic response (Dvornikov et al., 2016). However, contradictory results using peptides found that major conformational changes occur upon phosphorylation (Heller et al., 2003; Howarth et al., 2007). The fact that phosphorylation causes only subtle structural changes is in accordance with the fact that the Ca^2+^-sensitivity change upon phosphorylation is only 2-fold. It is interesting that despite these subtle changes, the physiological effect of phosphorylation is great.

Another exciting point arising from all these studies on troponin is its potential to determine how mutations affect troponin's structure, Ca^2+^ binding and also the effect of phosphorylation. Over 100 troponin mutations in cardiac TnT, TnC, or TnI have been linked so far with genetic cardiomyopathies. Different troponin mutations have different effects on protein-protein interactions, crossbridge cycle, myosin ATPase activity and phosphorylation levels of thin filament proteins, but all mutations affect troponin Ca^2+^-sensitivity (Marston, 2011; Lu et al., 2013). In a recent study it was observed that mutations in troponin causing cardiomyopathies lead to a decrease in the disorder score, reducing troponin's flexibility (Na et al., 2016).

Previous MD studies have been performed on how loss-of-function and gain-of-function mutations affect Ca^2+^ binding to troponin (Kekenes-Huskey et al., 2012; Lindert et al., 2012). Cheng et al. tried to explain how mutations affect phosphorylation changes using MD simulations in a study comparing non-phosphorylated and S23/24D phosphomimetic troponin containing the HCM mutations TnI R146G and R21C. Both mutations increased Ca^2+^-sensitivity and blunted the effect of phosphorylation, confirmed using biochemical methods. MD simulations showed that both mutations increased the Ca^2+^ binding affinity, as measured by distance between Ca^2+^ and Ser69 GO and inhibited formation of intrasubunit interactions between TnI N-terminus and inhibitory peptide in the phosphomimetic mutants, which are normally seen in S23/S24D WT troponin (Cheng et al., 2015). P83S mutation in the IT arm of TnI had similar effects, although the blunting effect was “weaker” in MD simulations (Cheng et al., 2016).

The same group studied the structural mechanism of TnI R145G HCM mutation that also blunts TnI phosphorylation (Regnier et al., 2014; Lindert et al., 2015). R145G increased the interaction between Ca^2+^ and Ser69 GO explaining the increased Ca^2+^ binding. On the other hand, these interactions were not observed in Zamora's analysis and the most evident effect of the mutations TnI R145G (HCM), TnI K36Q, and TnC G159D (both DCM) is that they trap Ser69 GO in the short distance configuration (Sheehan et al., 2016; Zamora et al., 2016b).

The changes caused by mutations can be reversible, as we recently discovered that Epigallocatechin-3-gallate (EGCG) and related compounds could restore the Ca^2+^-sensitivity change upon phosphorylation in DCM or HCM mutant thin filaments that were originally uncoupled (Papadaki et al., 2015). The molecular mechanism of action of EGCG is still to be elucidated, although docking studies indicate EGCG is likely to bind at the interface between N-terminus TnC and TnI (1–30). Current MD studies provide a basis for the investigation of this recoupling process (Hwang, 2016; Marston et al., 2016).

**Figure 2:**
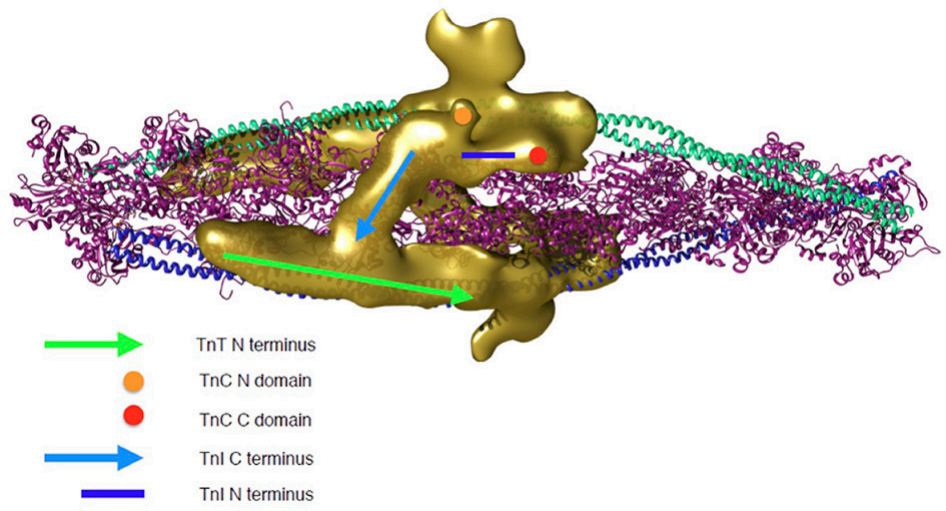
**Troponin rendering onto the thin filament**. Thin filament is represented by two coiled coil tropomyosin monomers shown in cyan and blue, decorated by actin monomers shown in ribbon view in magenta. Troponin's electron density as determined by 3D reconstruction from cryo-EM is represented in golden. Two troponin monomers are shown as they are predicted to be located on the thin filament and the different regulatory regions are indicated. Green arrow, N-terminus of TnT; light blue arrow, C-terminus of TnI (dynamic region); dark blue line, N-terminal extension of TnI (dynamic region); Orange circle, N-terminal domain of TnC; Red circle, C-terminal domain of TnC. The 3D reconstruction coordinates were kindly supplied by Dr William Lehman (Boston University, MA, USA) and the image was constructed by Mr Juan Eiros Zamora (Imperial College London, UK).

To address how troponin regulates muscle contractility it is necessary to extend studies from isolated troponin to the thin filament. Yang et al have made the most comprehensive reconstruction of actin-tropomyosin-troponin in the absence of Ca^2+^ at a resolution of 25Å and have located troponin on the previously determined actin-tropomyosin, showing details never observed before (Yang et al., 2014). Most strikingly, the C-terminal inhibitory domain of TnI is orientated across the filament, binding to actin close to the N-terminal TnT of the troponin on the opposite side of the filament. The density envelope corresponding to the core domain of troponin is apparent and an attempt has been made to fit troponin into this; the mobile parts of the molecule are, as expected, not seen but the orientation of troponin core indicates that both the regulatory Ca^2+^ binding site and the phosphorylatable serines of TnI would be accessible to the solution (Figure 2).

Of course this is a static model of just one state; a fully dynamic model of the thin filament is not yet accessible, although progress is being made on several fronts. The dynamics of the actin filament have been simulated using course-grained methods (Fan et al., 2012; Saunders and Voth, 2012) and the structure of tropomyosin alone and bound to actin has been simulated by MD (Zheng et al., 2013). Manning et al have developed the EM structures of the thin filament into a fully atomistic model and derived structures for the whole filament by energy minimization (Manning et al., 2011). This method seems capable of reproducing the features of the Ca^2+^ switch and even to explain how mutations like TnT R92Q that are remote from troponin can change Ca^2+^-sensitivity by using MD simulations of the whole thin filament (Williams et al., 2016). However, current MD simulations are run for less than 5 ns and so do not even start to explore the range of conformations possible in real life.

## Conclusions

In conclusion, troponin has a rather dynamic structure, owing to the intrinsically disordered domains. This brings difficulties in elucidating its exact structure and thus the mechanisms of its regulation. Determining the structure of troponin mobile regions will not only lead to understanding the changes that occur upon its regulation, but also to predicting the effect of a mutation or a pharmacological agent. For example, just by knowing the structure of tropomyosin and its exact interactions with actin it has been possible to predict the effect of disease-causing mutations (Marston et al., 2013; Donkervoort et al., 2015). With troponin it is not possible to make such predictions at the moment, as the structure is still unresolved. However, MD simulations may be the best way to study troponin, since the molecule is treated as a moving object and not as a rigid structure. Technical and mathematical advances give hope that in the near future it will be possible to simulate an ensemble of several million atoms for μs and thereby finally get a full description of the dynamic regulated thin filament.

## Author contributions

MP wrote the manuscript and SM edited it.

## Funding

This work has been supported by the British Heart Foundation (RG/11/20/29266 and FS/12/24/29568).

### Conflict of interest statement

The authors declare that the research was conducted in the absence of any commercial or financial relationships that could be construed as a potential conflict of interest.
